# Supplementary material for: Grain versus AIN: Common rodent diets differentially affect health outcomes in adult C57BL/6j mice
Source: PLoS One. 2024 Mar 21;19(3):e0293487. doi: 10.1371/journal.pone.0293487 (PMC10956799; doi:10.1371/journal.pone.0293487)

Supplementary Figure 1. Flow diagram of study. A) Flow diagram of study for female mice. B) Flow diagram of study for male mice. Grain: grain-based diet; Syn: semi-synthetic diet.

Supplementary Figure 1A Flow diagram of study for females.

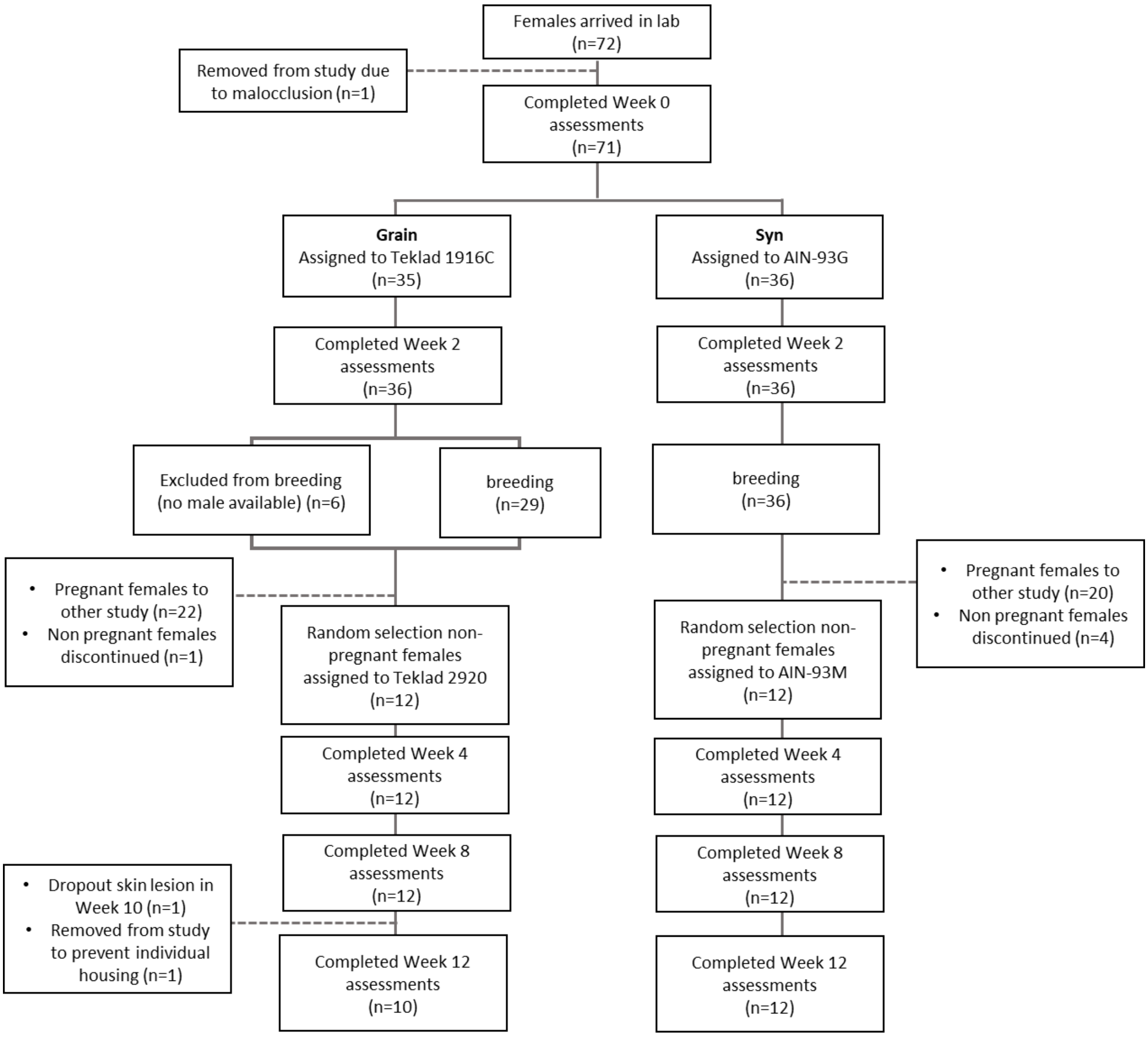

Supplementary Figure 1B. **Flow diagram of study for males.**

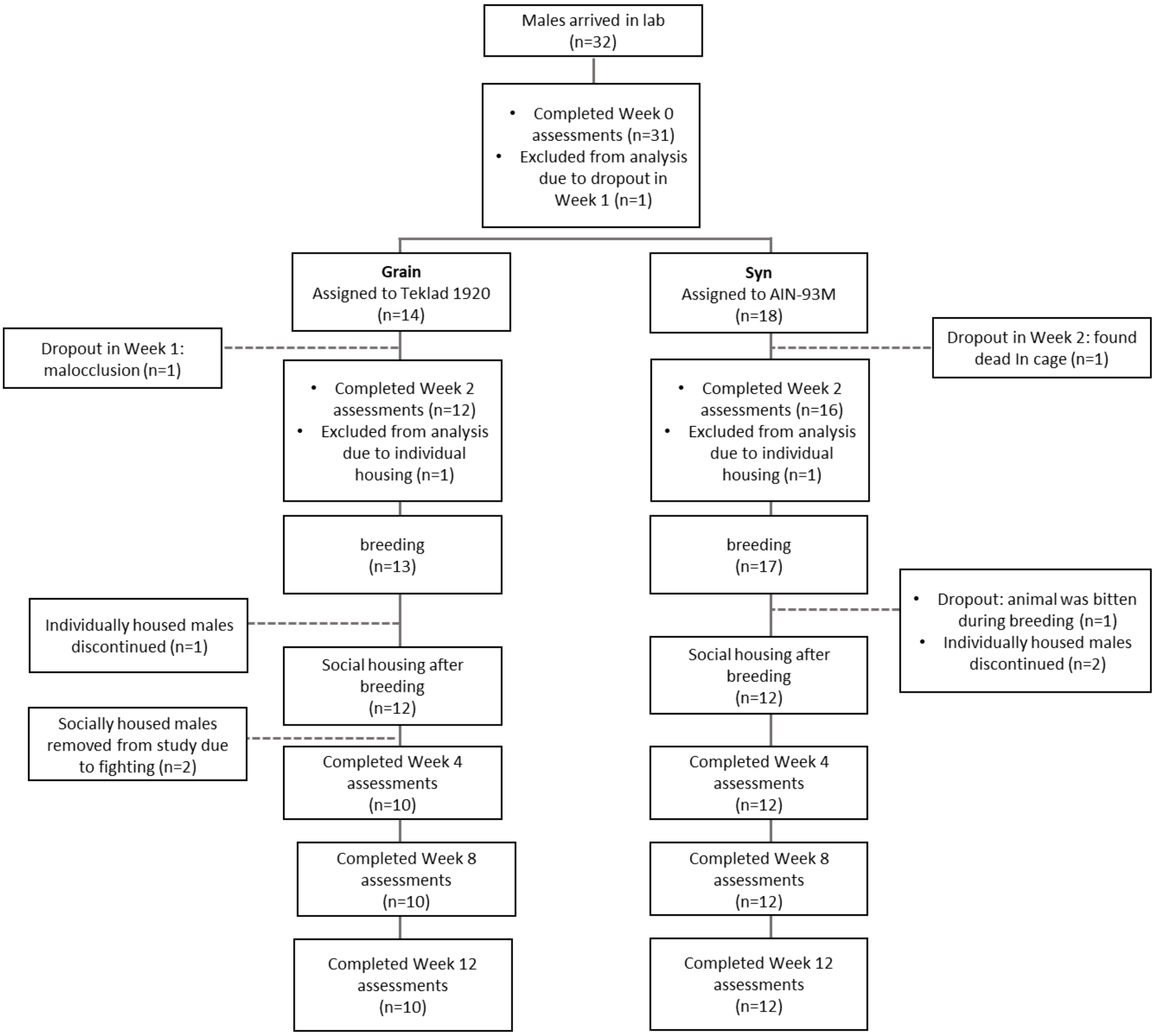

Supplement: S1 Fig — A) Flow diagram of study for female mice. B) Flow diagram of study for male mice. Grain: grain-based diet; Syn: semi-synthetic diet. (PDF) [file pone.0293487.s001.pdf]
