# Supplementary material for: Grain versus AIN: Common rodent diets differentially affect health outcomes in adult C57BL/6j mice
Source: PLoS One. 2024 Mar 21;19(3):e0293487. doi: 10.1371/journal.pone.0293487 (PMC10956799; doi:10.1371/journal.pone.0293487)
Supplement: S2 Fig — A) Alpha-diversity of female mice fed Grain (n = 10 – 13a) or Syn (n = 8 – 14a) diet assessed by Chao1 index. B) Beta-diversity assessed by principle coordinate analysis (PCoA), using Bray-Curtis distance metrics. C-O) Box plots of bacterial taxa (at genus level) at week 0 and week 2 with significant interaction, as assessed with generalized linear models with mixed effects on the sequencing counts followed by Chi Squared test. The resulting p-values were corrected using Benjamini-Hochberg. Data presented as median ± interquartile range. *p < 0.05, a fecal samples were not collected when mice did not defecate voluntarily at the time of collection. Grain: grain-based diet; Syn: semi-synthetic diet. (PDF) [file pone.0293487.s002.pdf]

Supplementary Figure 2.

Fecal microbiota composition analyses in female mice at week 0 and week 2. A) Alpha-diversity of female mice fed Grain (n = 10 – 13[a]) or Syn (n = 8 – 14[a]) diet assessed by Chao1 index. B) Beta-diversity assessed by principle coordinate analysis (PCoA), using Bray-Curtis distance metrics. C-O) Box plots of bacterial taxa (at genus level) at week 0 and week 2 with significant interaction, as assessed with generalized linear models with mixed effects on the sequencing counts followed by Chi Squared test. The resulting p-values were corrected using Benjamini-Hochberg. Data presented as median ± interquartile range. *$p < 0.05$, [a] fecal samples were not collected when mice did not defecate voluntarily at the time of collection. Grain: grain-based diet; Syn: semi-synthetic diet.

A

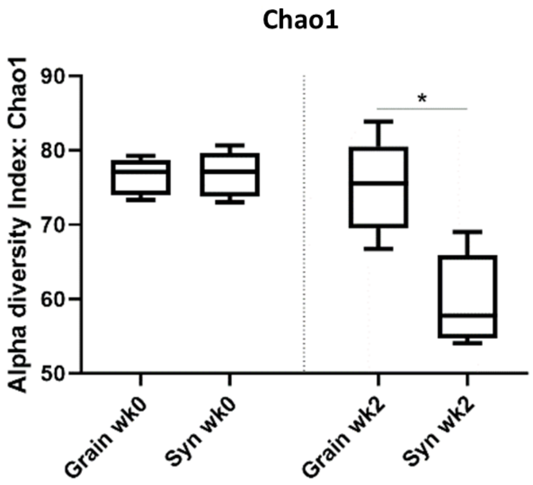

B

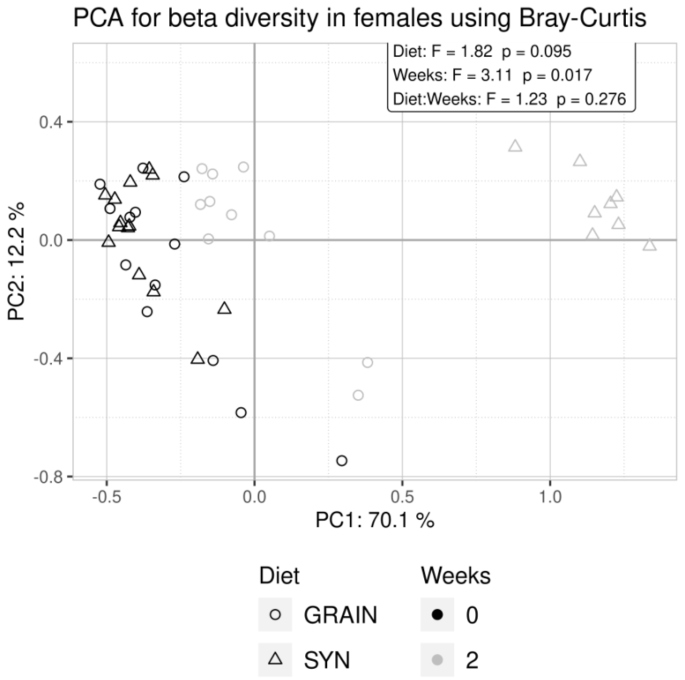

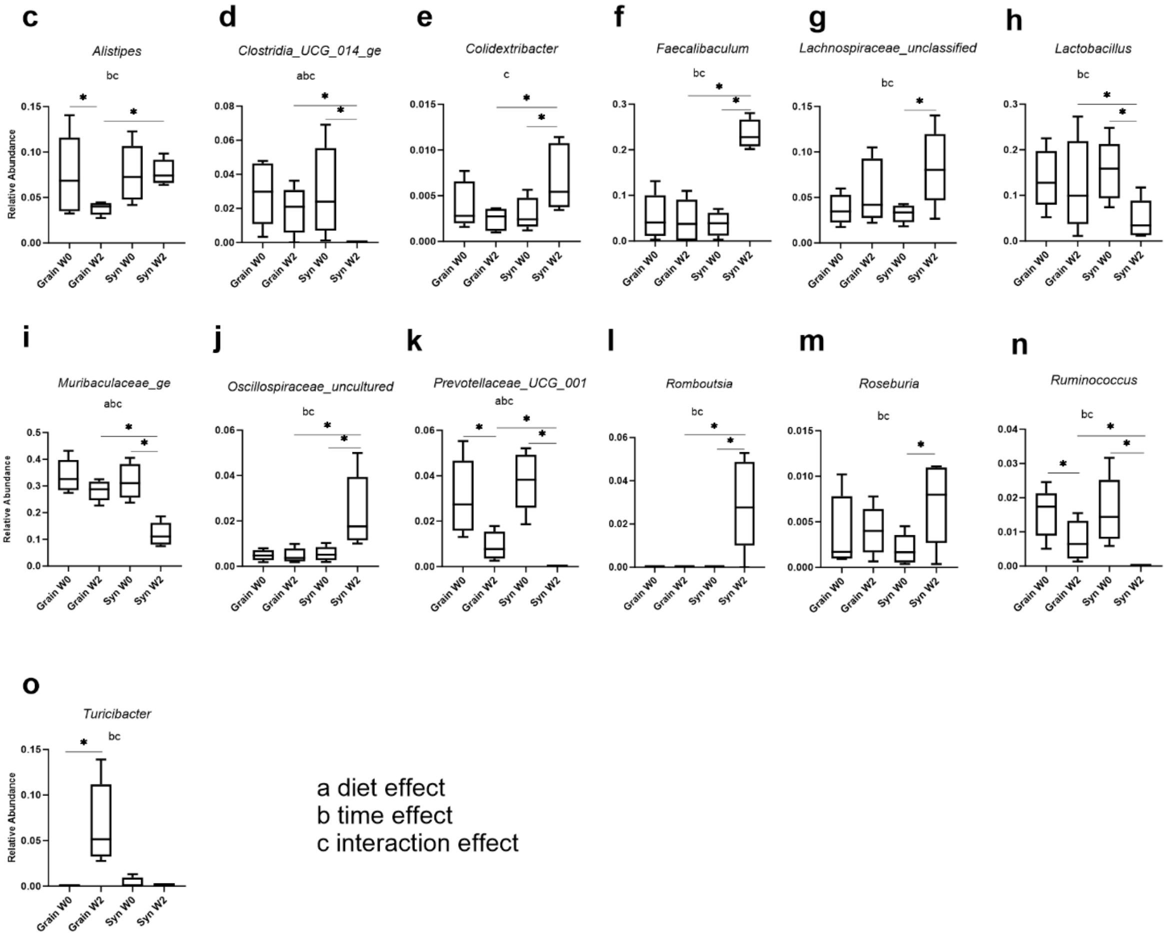
c
Alistipes
bc
Relative Abundance
Grain W0
Grain W2
Syn W0
Syn W2
d
Clostridia_UCG_014_ge
abc
e
Colidextribacter
c
f
Faecalibaculum
bc
g
Lachnospiraceae_unclassified
bc
h
Lactobacillus
bc
i
Muribaculaceae_ge
abc
j
Oscillospiraceae_uncultured
bc
k
Prevotellaceae_UCG_001
abc
l
Romboutsia
bc
m
Roseburia
bc
n
Ruminococcus
bc
o
Turicibacter
bc
a diet effect
b time effect
c interaction effect
