# Supplementary material for: Grain versus AIN: Common rodent diets differentially affect health outcomes in adult C57BL/6j mice
Source: PLoS One. 2024 Mar 21;19(3):e0293487. doi: 10.1371/journal.pone.0293487 (PMC10956799; doi:10.1371/journal.pone.0293487)
Supplement: S3 Fig — A-D) Changes in body weight and body composition of male mice on Grain, (n = 12) or semi-synthetic diet (Syn, n = 16). E. Alpha-diversity of male mice fed Grain (n = 5 – 14a) or Syn (n = 3 – 12a) diet assessed by Chao1 index. F) Beta-diversity assessed by principle coordinate analysis (PCoA), using Bray-Curtis distance metrics. G) Box plots of bacterial taxa (at genus level) at week 0 and week 2 with significant interaction, as assessed with generalized linear models with mixed effects on the sequencing counts followed by Chi Squared test. The resulting p-values were corrected using Benjamini-Hochberg. Data presented as median ± interquartile range. *p < 0.05, a fecal samples were not collected when mice did not defecate voluntarily at the time of collection Grain: grain-based diet; Syn: semi-synthetic diet. (PDF) [file pone.0293487.s003.pdf]

Supplementary Figure 3.

Change in body composition in male mice over two week experimental timeline and fecal microbiota composition analyses in male mice at week 0 and week 2. A-D) Changes in body weight and body composition of male mice on Grain, (n = 12) or semi-synthetic diet (Syn, n = 16). E. Alpha-diversity of male mice fed Grain (n = 5 – 14[a]) or Syn (n = 3 – 12[a]) diet assessed by Chao1 index. F) Beta-diversity assessed by principle coordinate analysis (PCoA), using Bray-Curtis distance metrics. G) Box plots of bacterial taxa (at genus level) at week 0 and week 2 with significant interaction, as assessed with generalized linear models with mixed effects on the sequencing counts followed by Chi Squared test. The resulting p-values were corrected using Benjamini-Hochberg. Data presented as median ± interquartile range. *$p < 0.05$, [a] fecal samples were not collected when mice did not defecate voluntarily at the time of collection Grain: grain-based diet; Syn: semi-synthetic diet.

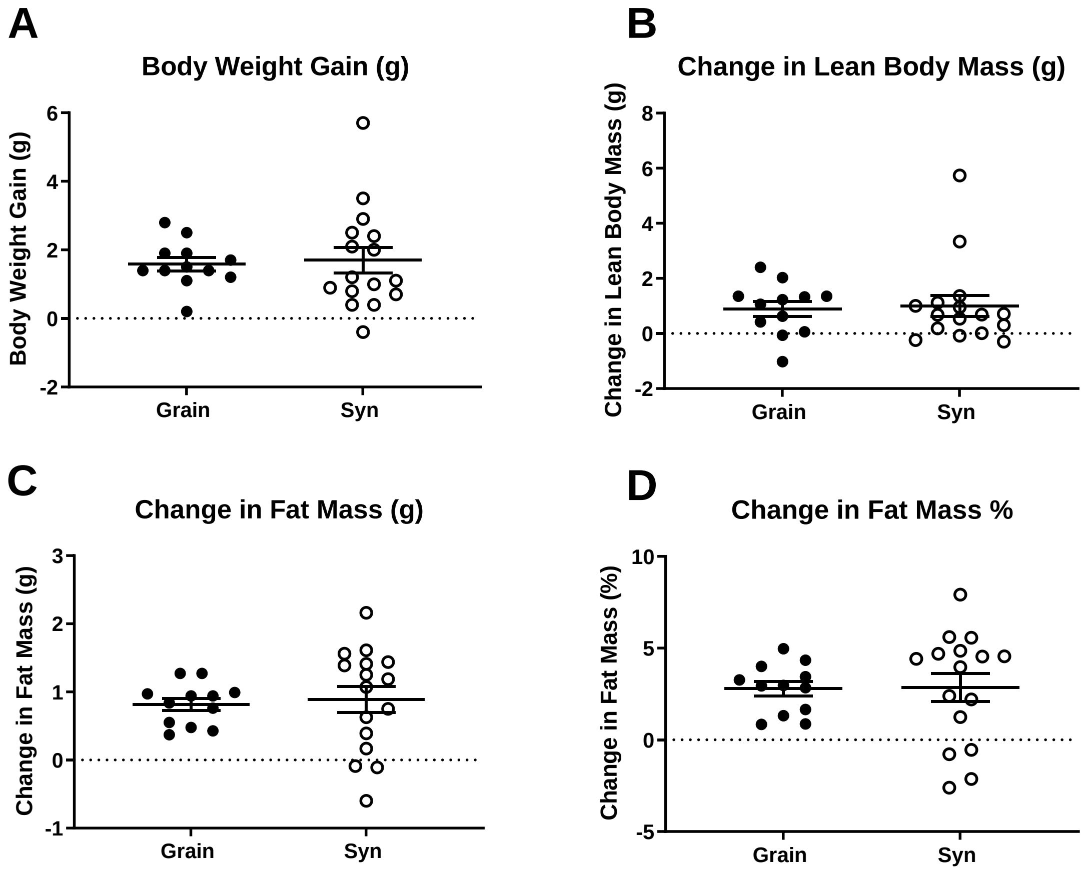

E

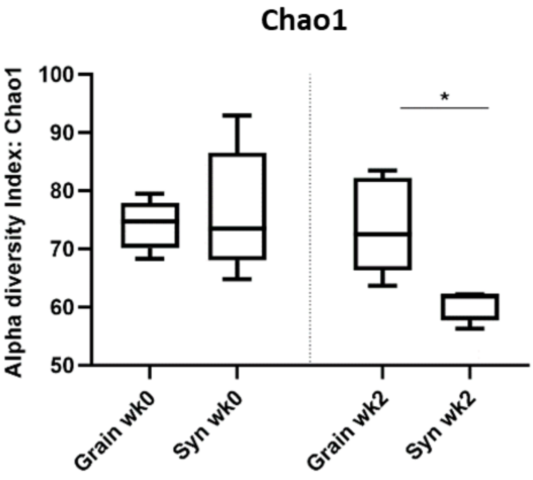

F

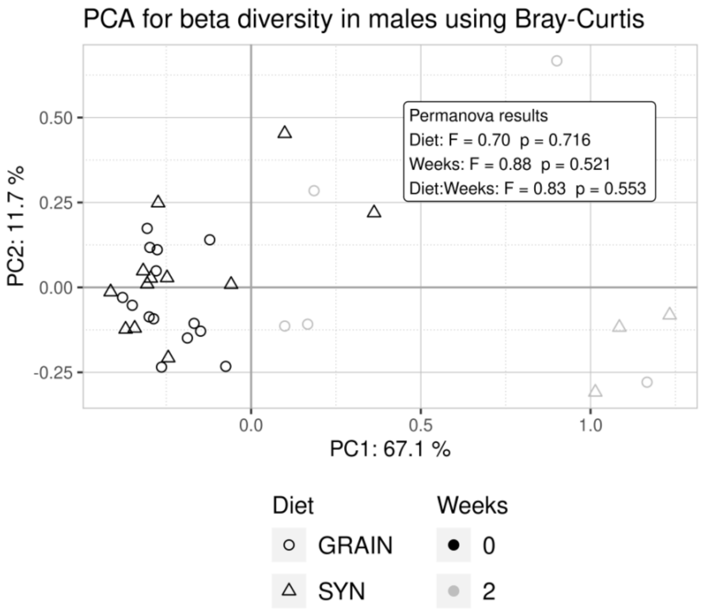

G

Genera	Diet Chi	Diet P(Chi)	Diet p-adjust	Weeks Chi	Weeks P(Chi)	Weeks p-adjust	Interaction Chi	Interaction P(Chi)	Interactic p-adjust
**Clostridia_UCG_014_ge**	7.217	0.007	0.087	13.883	0	0	24.084	0	0
**Muribaculaceae_ge**	0.938	0.333	0.711	62.776	0	0	26.965	0	0
**Parasutterella**	2.17	0.141	0.487	8.903	0.003	0.005	16.476	0	0
**Prevotellaceae_UCG_001**	11.361	0.001	0.018	117.329	0	0	33.682	0	0
**Ruminococcus**	2.156	0.142	0.487	28.147	0	0	25.213	0	0
