# Supplementary material for: Grain versus AIN: Common rodent diets differentially affect health outcomes in adult C57BL/6j mice
Source: PLoS One. 2024 Mar 21;19(3):e0293487. doi: 10.1371/journal.pone.0293487 (PMC10956799; doi:10.1371/journal.pone.0293487)
Supplement: S4 Fig — Summary of body weight, body composition and dissected organ weights of male mice on Grain (n = 10) or semi-synthetic diet (Syn, n = 12) from arrival to 12 weeks. A) Changes in body weight. B) Changes in lean body mass. C) Changes in fat mass. D) Changes in percentage fat mass. E) Dissected organ weights at 12 weeks of age. Data are mean ± SEM. * p < 0.05, # 0.05 < p < 0.06. BW: body weight; BAT: brown adipose tissue; WAT: white adipose tissue; SCWAT: subcutaneous WAT; ViscWAT: visceral WAT; TG: triglycerides; BCA: bicinchoninic acid; Grain: grain-based diet; Syn: semi-synthetic diet. (PDF) [file pone.0293487.s004.pdf]

Supplementary Figure 4.

Changes in body composition of male mice during 12 week experimental timeline. Summary of Summary of body weight, body composition and dissected organ weights of male mice on Grain (n = 10) or semi-synthetic diet (Syn, n = 12) from arrival to 12 weeks. A) Changes in body weight. B) Changes in lean body mass. C) Changes in fat mass. D) Changes in percentage fat mass. E) Dissected organ weights at 12 weeks of age. Data are mean ± SEM. * $p < 0.05$, $^{\#}$ $0.05 < p < 0.06$. BW: body weight; BAT: brown adipose tissue; WAT: white adipose tissue; SCWAT: subcutaneous WAT; ViscWAT: visceral WAT; TG: triglycerides; BCA: bicinchoninic acid; Grain: grain-based diet; Syn: semi-synthetic diet.

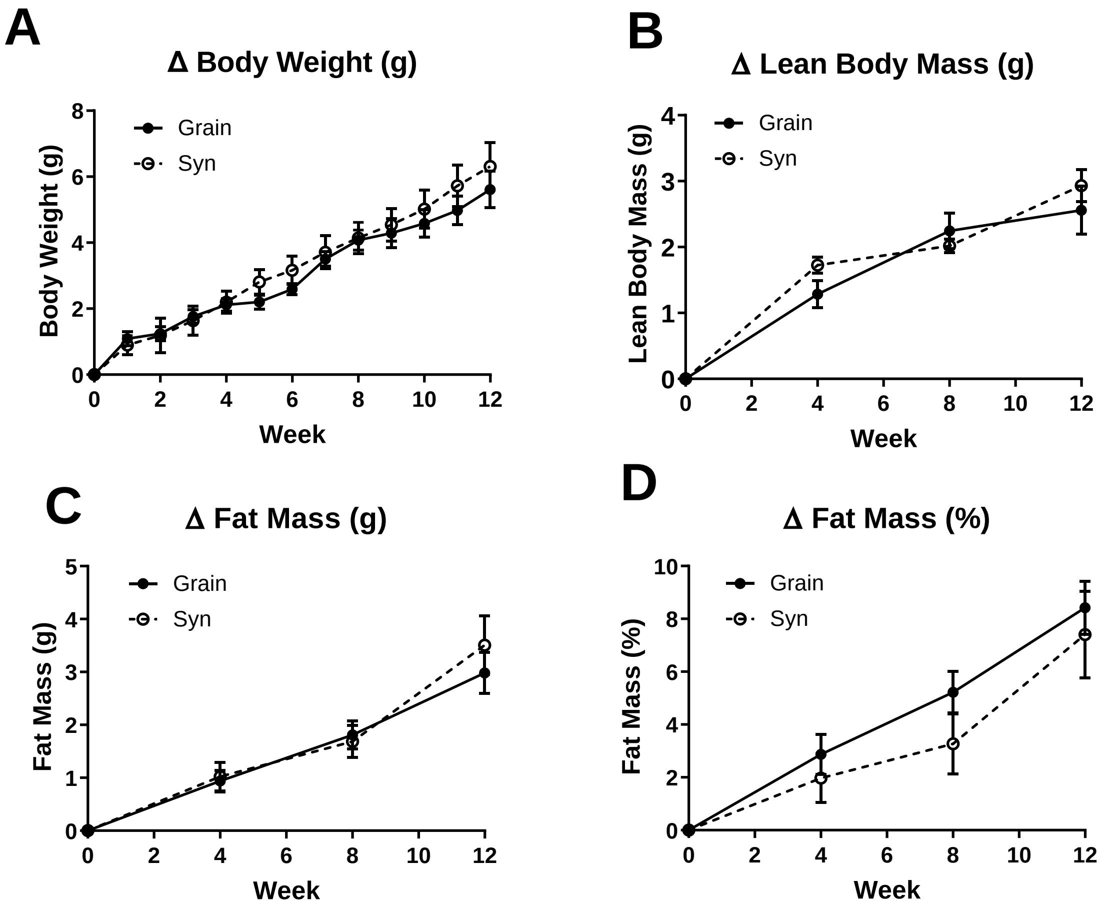

E

	Grain (n = 10)	Syn (n = 12)
**Body weight (g)**	28.9 ± 0.36	31.0 ± 0.46*
**BAT (% BW)**	0.50 ± 0.03	0.54 ± 0.04
**Total WAT (% BW)**	3.98 ± 0.27	4.60 ± 0.46
**SCWAT (% BW)**	1.10 ± 0.06	1.30 ± 0.10

**ViscWAT (% BW)**	2.88 ± 0.22	3.29 ± 0.22
Gonadal (% BW)	2.07 ± 0.15	2.35 ± 0.27
Retroperitoneal (% BW)	0.64 ± 0.06	0.74 ± 0.08
Perirenal (% BW)	0.18 ± 0.02	0.20 ± 0.03
**Liver (% BW)**	4.64 ± 0.06	4.02 ± 0.12*
Liver TG/BCA protein (mg)	0.10 ± 0.02	0.18 ± 0.03[#]
**Adrenals (% BW)**	0.01 ± 0.01	0.01 ± 0.01
Plasma corticosterone (ng/ml)	11.1 ± 2.05	12.5 ± 4.31
**Thymus (% BW)**	0.16 ± 0.01	0.17 ± 0.15
