# Supplementary material for: Grain versus AIN: Common rodent diets differentially affect health outcomes in adult C57BL/6j mice
Source: PLoS One. 2024 Mar 21;19(3):e0293487. doi: 10.1371/journal.pone.0293487 (PMC10956799; doi:10.1371/journal.pone.0293487)
Supplement: S5 Fig — A-O) Boxplots of bacterial taxa (at genus level) of female mice fed Grain (n = 8 – 12a) or Syn (n = 7 – 12a) diet with significant interactions, as assessed with generalized linear models with mixed effects on the sequencing counts followed by Chi Squared test. The resulting p-values were corrected using Benjamini-Hochberg. Data presented as median ± interquartile range. * p < 0.05. a fecal samples were not collected when mice did not defecate voluntarily at the time of collection. Grain: grain-based diet; Syn: semi-synthetic diet. (PDF) [file pone.0293487.s005.pdf]

Supplementary Figure 5.

Fecal microbiota composition analyses in female mice at week 0 and week 12. A-O) Boxplots of bacterial taxa (at genus level) of female mice fed Grain (n = 8 – 12[a]) or Syn (n = 7 – 12[a]) diet with significant interactions, as assessed with generalized linear models with mixed effects on the sequencing counts followed by Chi Squared test. The resulting p-values were corrected using Benjamini-Hochberg. Data presented as median ± interquartile range. * p < 0.05. [a] fecal samples were not collected when mice did not defecate voluntarily at the time of collection. Grain: grain-based diet; Syn: semi-synthetic diet.

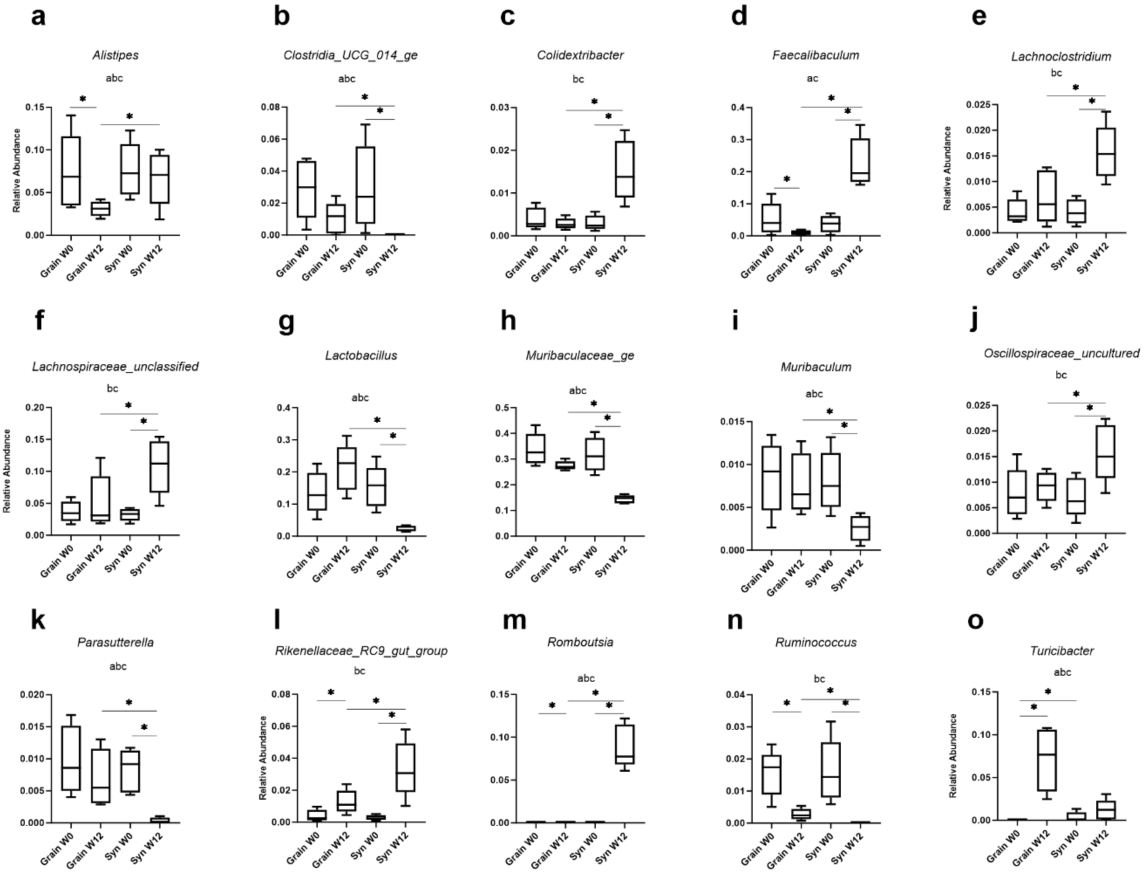

a diet effect
b time effect
c interaction effect
