# Supplementary material for: Grain versus AIN: Common rodent diets differentially affect health outcomes in adult C57BL/6j mice
Source: PLoS One. 2024 Mar 21;19(3):e0293487. doi: 10.1371/journal.pone.0293487 (PMC10956799; doi:10.1371/journal.pone.0293487)
Supplement: S1 Table — A) for female mice. B) for male mice. Data are mean ± SEM. Differences between batches are considered significant when p < 0.05. a significantly different compared to batch 1; b significantly different compared to batch 2. Grain: grain-based diet; Syn: semi-synthetic diet. (PDF) [file pone.0293487.s007.pdf]

Supplementary Table 1

Body weight and body composition of animals per batch at arrival and after two weeks. A) for female mice. B) for male mice. Data are mean ± SEM. Differences between batches are considered significant when $p < 0.05$. [a] significantly different compared to batch 1; [b] significantly different compared to batch 2. Grain: grain-based diet; Syn: semi-synthetic diet.

Supplementary Table 1A Females

Females	Total	Batch 1	Batch 2	Batch 3	Batch 4
			**Number of animals (n)**		
Week 0 (All)	71	20	24	21	6
Week 0 (Syn)	36	12	16	8	
Week 0 (Grain)	35	8	8	13	6
Week 2 (Syn)	36	12	16	8	
Week 2 (Grain)	35	8	8	13	6
			**Body weight**		
Week 0 (all)	19.8 ± 0.12	19.3 ± 0.21	20.4 ± 0.16[a]	19.6 ± 0.21[b]	19.5 ± 0.49[b]
Week 0 (Syn)	19.9 ± 0.16	19.4 ± 0.28	20.5 ± 0.19[a]	19.6 ± 0.21[b]	
Week 0 (Grain)	19.7 ± 0.18	19.3 ± 0.35	20.2 ± 0.31	19.7 ± 0.32	19.5 ± 0.49
Week 2 (Syn)		20.0 ± 0.23	19.5 ± 0.21	20.1 ± 0.24	
Week 2 (Grain)		20.1 ± 0.36	20.0 ± 0.27	20.0 ± 0.32	19.9 ± 0.15
			**Fat mass (g)**		
Week 0 (all)	2.94 ± 0.06	2.85 ± 0.08	3.17 ± 0.15[a]	2.79 ± 0.06[b]	2.82 ± 0.14
Week 0 (Syn)	2.95 ± 0.06	2.86 ± 0.11	3.10 ± 0.08	2.81 ± 0.09	
Week 0 (Grain)	2.93 ± 0.11	2.85 ± 0.13	3.33 ± 0.43	2.77 ± 0.09	2.82 ± 0.14
Week 2 (Syn)		2.07 ± 0.08	2.23 ± 0.15	2.21 ± 0.10	
Week 2 (Grain)		2.28 ± 0.15	2.62 ± 0.27	2.27 ± 0.18	2.78 ± 0.30
			**Lean body mass (g)**		
Week 0 (All)	15.8 ± 0.11	15.7 ± 0.18	15.9 ± 0.20	15.9 ± 0.19	15.8 ± 0.45
Week 0 (Syn)	15.8 ± 0.12	15.8 ± 0.24	16.0 ± 0.19	15.6 ± 0.18	
Week 0 (Grain)	15.8 ± 0.18	15.7 ± 0.29	15.6 ± 0.46	16.0 ± 0.29	15.8 ± 0.45
Week 2 (Syn)		17.0 ± 0.20	16.3 ± 0.21[a]	16.8 ± 0.24	0.07
Week 2 (Grain)		17.0 ± 0.24	16.4 ± 0.35	16.6 ± 0.25	16.4 ± 0.34

			Percent fat mass (%)		
Week 0 (All)	14.8 ± 0.28	14.8 ± 0.37	15.6 ± 0.70	14.2 ± 0.26	14.5 ± 0.75
Week 0 (Syn)	14.8 ± 0.26	14.8 ± 0.49	15.2 ± 0.41	14.3 ± 0.37	
Week 0 (Grain)	14.8 ± 0.51	14.8 ± 0.62	16.4 ± 0.01	14.1 ± 0.35	14.5 ± 0.75
Week 2 (Syn)		10.4 ± 0.40	11.5 ± 0.74	11.0 ± 0.44	
Week 2 (Grain)		11.3 ± 0.57	13.1 ± 1.30	11.3 ± 0.82	13.9 ± 1.38

Supplementary Table 1B Males

Males	Total	Batch 1	Batch 2	Batch 3
		**Number of animals (n)**		
Week 0 (all)	31	10	11	10
Week 0 (Syn)	17	6	7	4
Week 0 (Grain)	14	4	4	6
Week 2 (Syn)	14	6	6	4
Week 2 (Grain)	12	2	4	6
		**Body weight (g)**		
Week 0 (All)	24.1 ± 0.31	25.0 ± 0.38	24.5 ± 0.61	22.8 ± 0.33[a,b]
Week 0 (Syn)	24.5 ± 0.41	24.4 ± 0.46	25.3 ± 0.74	23.2 ± 0.60
Week 0 (Grain)	23.7 ± 0.47	25.9 ± 0.35	23.2 ± 0.78[a]	22.5 ± 0.39[a]
Week 2 (Syn)		26.0 ± 0.71	26.4 ± 0.57	26.6 ± 1.14
Week 2 (Grain)		28.2 ± 0.01	25.0 ± 0.82[a]	23.9 ± 0.57[a]
		**Fat (g)**		
Week 0 (All)	2.10 ± 0.05	2.10 ± 0.06	2.12 ± 0.10	2.09 ± 0.10
Week 0 (Syn)	2.17 ± 0.06	2.15 ± 0.08	2.10 ± 0.10	2.33 ± 0.14
Week 0 (Grain)	2.02 ± 0.08	2.03 ± 0.08	2.16 ± 0.25	1.93 ± 0.09
Week 2 (Syn)		3.27 ± 0.25	2.73 ± 0.30	3.29 ± 0.44
Week 2 (Grain)		2.46 ± 0.04	2.87 ± 0.28	2.94 ± 0.11
		**Lean body mass (g)**		
Week 0 (all)	21.0 ± 0.29	22.0 ± 0.39	21.1 ± 0.59	20.0 ± 0.26[a]
Week 0 (Syn)	21.3 ± 0.39	21.4 ± 0.49	21.9 ± 0.73	20.1 ± 0.53
Week 0 (Grain)	20.7 ± 0.44	22.9 ± 0.35	19.8 ± 0.60[a]	19.9 ± 0.31[a]
Week 2 (Syn)		21.9 ± 0.66	23.0 ± 0.72	22.4 ± 1.60
Week 2 (Grain)		25.0 ± 0.02	21.3 ± 0.58[a]	20.1 ± 0.47[a]
		**Fat (%)**		
Week 0 (All)	8.75 ± 0.21	8.44 ± 0.30	8.69 ± 0.43	9.15 ± 0.36
Week 0 (Syn)	8.93 ± 0.31	8.83 ± 0.40	8.37 ± 0.48	10.1 ± 0.54
Week 0 (Grain)	8.54 ± 0.30	7.86 ± 0.32	9.24 ± 0.84	8.53 ± 0.29

Week 2 (Syn)	12.6 ± 1.01	10.4 ± 1.30	12.7 ± 2.21
Week 2 (Grain)	8.72 ± 0.11	11.4 ± 0.81[a]	12.3 ± 0.29[a]
