# Supplementary material for: Grain versus AIN: Common rodent diets differentially affect health outcomes in adult C57BL/6j mice
Source: PLoS One. 2024 Mar 21;19(3):e0293487. doi: 10.1371/journal.pone.0293487 (PMC10956799; doi:10.1371/journal.pone.0293487)
Supplement: S2 Table — A) for female mice. B) for male mice. Average caloric intake was calculated per animal per day based on weekly weight of food consumed per cage. Data are mean ± SEM. * p < 0.05. Grain: grain-based diet; Syn: semi-synthetic diet. (PDF) [file pone.0293487.s008.pdf]

Supplementary Table 2

Estimate of average daily caloric intake of mice during acclimatization and throughout the study (per four week interval). A) for female mice. B) for male mice. Average caloric intake was calculated per animal per day based on weekly weight of food consumed per cage. Data are mean ± SEM. * $p < 0.05$. Grain: grain-based diet; Syn: semi-synthetic diet.

Supplementary Table 2A. Estimate of average daily caloric intake in female mice

**Females**	**Grain** (n = 18 cages)	**Syn** (n = 18 cages)
Acclimatization (0 to 2 weeks)	8.46 ± 0.14	9.96 ± 0.11*
	**Grain** (n = 6 cages)	**Syn** (n = 5 - 6 cages)
0 to 4 weeks	8.50 ± 0.29	10.32 ± 0.12*
4 to 8 weeks	9.17 ± 0.19	10.98 ± 0.34*
8 to 12 weeks	8.87 ± 0.75	10.18 ± 0.26

Supplementary Table 2B. Estimate of average daily caloric intake in male mice

**Males**	**Grain** (n = 7 cages)	**Syn** (n = 6 cages)
Acclimatization (0 to 2 weeks)	11.06 ± 0.54	11.16 ± 0.60
	**Grain** (n = 6 cages)	**Syn** (n = 5 cages)
0 to 4 weeks	10.98 ± 0.50	10.95 ± 0.40
4 to 8 weeks	10.52 ± 1.05	11.41 ± 0.62
8 to 12 weeks	9.27 ± 0.24	11.28 ± 0.46*
