# Supplementary material for: Grain versus AIN: Common rodent diets differentially affect health outcomes in adult C57BL/6j mice
Source: PLoS One. 2024 Mar 21;19(3):e0293487. doi: 10.1371/journal.pone.0293487 (PMC10956799; doi:10.1371/journal.pone.0293487)
Supplement: S3 Table — Grain: grain-based diet; Syn: semi-synthetic diet. (PDF) [file pone.0293487.s009.pdf]

Supplementary Table 3.

Body composition of Grain and Syn-fed pregnant and non-pregnant females at mating. Grain: grain-based diet; Syn: semi-synthetic diet.

	**Grain pregnant (n = 22)**	**Grain non-pregnant (n = 7)**	**Syn pregnant (n = 20)**	**Syn non-pregnant (n = 16)**
**Body weight (g)**	20.0 ± 0.20	20.0 ± 0.49	19.6 ± 0.17	20.0 ± 0.22
**Lean body mass (g)**	16.7 ± 0.17	16.8 ± 0.42	16.5 ± 0.15	16.8 ± 0.22
**Fat mass (g)**	2.42 ± 0.15	2.76 ± 0.42	2.18 ± 0.09	2.17 ± 0.13
**Fat mass (%)**	12.1 ± 0.68	11.8 ± 0.54	11.1 ± 0.44	10.9 ± 0.63
