# Supplementary material for: Grain versus AIN: Common rodent diets differentially affect health outcomes in adult C57BL/6j mice
Source: PLoS One. 2024 Mar 21;19(3):e0293487. doi: 10.1371/journal.pone.0293487 (PMC10956799; doi:10.1371/journal.pone.0293487)
Supplement: S4 Table — Short chain fatty acid (SCFA) measurements in cecal content of Grain (n = 8a) or semi-synthetic diet (Syn, n = 9a) male mice. Data are mean ± SEM. * p < 0.05, # 0.05 < p < 0.06. a upon dissection cecums were not collected for 2 animals in Syn and one animal in the Grain diet groups, and for SCFA one cecal sample per diet group was lost. Grain: grain-based diet; Syn: semi-synthetic diet. (PDF) [file pone.0293487.s010.pdf]

Supplementary Table 4

Cecal short chain fatty acid (SCFA) of male mice

Short chain fatty acid (SCFA) measurements in cecal content of Grain (n = 8[a]) or semi-synthetic diet (Syn, n = 9[a]) male mice. Data are mean ± SEM. * $p < 0.05$, # $0.05 < p < 0.06$. [a] upon dissection cecums were not collected for 2 animals in Syn and one animal in the Grain diet groups, and for SCFA one cecal sample per diet group was lost. Grain: grain-based diet; Syn: semi-synthetic diet.

	**Grain (n = 8)**	**Syn (n = 9)**
**Content (% of body weight)**	0.80 ± 0.05	0.59 ± 0.07
**Total SCFA (mmol/L)**	71.7 ± 5.83	42.7 ± 5.18*
Acetic acid (% of total)	69.1 ± 0.88	77.0 ± 1.67*
Butyric acid (% of total)	17.5 ± 1.61	9.71 ± 1.36*
Propionic acid (% of total)	13.4 ± 0.80	14.0 ± 0.75
Valeric acid (% of total)	1.34 ± 0.08	1.63 ± 0.09[#]
**Acetic:Propionic ratio**	5.25 ± 0.28	5.58 ± 0.38
**Acetic:Butyric ratio**	4.21 ± 0.43	8.86 ± 0.96*
