# Supplementary material for: Grain versus AIN: Common rodent diets differentially affect health outcomes in adult C57BL/6j mice
Source: PLoS One. 2024 Mar 21;19(3):e0293487. doi: 10.1371/journal.pone.0293487 (PMC10956799; doi:10.1371/journal.pone.0293487)
Supplement: S1 File — (PDF) [file pone.0293487.s011.pdf]

Supplementary File 1

Descriptive results about incidence of fighting in male mice social housing after breeding.

The male mice that were used is this study were pair housed after arrival with a same-sex sibling. After the acclimatization period breeding commenced, during which each male was housed for 72 hours with 2 females. In total 12 male pairs (7 pairs on Syn and 5 pairs on Grain diet) were re-housed with the same male cage mate after breeding. Fighting incidence was closely monitored in the week following breeding and animals were checked twice daily for bite marks or other injuries. Fighting was detected on one occasion resulting in one animal (Syn diet) with a bite wound on the day directly following reuniting, the pair was immediately separated. The remaining 11 pairs were kept under the same housing conditions for the remainder of the experiment. No excessive fighting was observed nor were injuries or scars indicative of fighting detected at any timepoint during the study. No statistical analysis was performed on these data. Grain: grain-based diet; Syn: semi-synthetic diet.
